# Association between intraoperative fluoroscopy for anterior approach total hip arthroplasty and short-term revision risk: an analysis based on 49,878 cases from the Dutch Arthroplasty Register

**DOI:** 10.2340/17453674.2026.45966

**Published:** 2026-06-22

**Authors:** Marije C VINK, Wierd P ZIJLSTRA, Liza N VAN STEENBERGEN, Paulien A VAN VEEN, Peter F DOORN, Jesse W P KUIPER, Rinne M PETERS

**Affiliations:** 1 Department of Orthopedic Surgery, Frisius Medical Center, Leeuwarden; 2 Department of Orthopedic Surgery, University Medical Center Groningen; 3 Dutch Arthroplasty Register (LROI), ‘s Hertogenbosch; 4 Department of Orthopedic Surgery, Martini Hospital, Groningen, the Netherlands; 5 Department of Orthopedic Surgery, Flinders Medical Center, Adelaide, Australia

## Abstract

**Background and purpose:**

Intraoperative fluoroscopy during primary total hip arthroplasty (THA) can be used to assist in component positioning in order to optimize placement and restore hip kinematics. Whether fluoroscopy leads to superior outcomes is subject to debate. We aimed to examine the use of fluoroscopy in the Netherlands and determine the association between the use of fluoroscopy and short-term revision risk following primary THA via the direct anterior approach (DAA).

**Methods:**

We included 49,878 primary THAs, performed via DAA, registered in the LROI from 2022–2024. Competing risk analysis and multivariable Cox-regression analyses were used to assess differences in implant survival between use of fluoroscopy and conventional surgery. Hazard ratios (HR) were adjusted for body mass index, previous surgeries, and fixation, and stratified for age, ASA class, and diagnosis.

**Results:**

Unadjusted cumulative incidence of revision after 6 months, 1, 2, and 2.5 years did not show significant differences: the 2.5-year revision rate was 1.7% (95% confidence interval [CI] 1.5–1.9) for the fluoroscopy group, and 2.0% (CI 1.8–2.3) in the conventional group. Multivariable analysis demonstrated that the use of fluoroscopy was associated with a significantly lower risk of revision than conventional surgery (HR 0.8, CI 0.7–0.9).

**Conclusion:**

The use of intraoperative fluoroscopy in primary THA via DAA in the Netherlands is associated with a significantly lower short-term risk of revision. The data showed no major differences in revision due to malalignment and periprosthetic fractures. Revision rates were low in both groups and clinical differences were only small.

Intraoperative fluoroscopy in primary total hip arthroplasty (THA) offers real-time feedback on implant positioning and is widely accessible. Fluoroscopy provides the surgeon with direct feedback on implant placement, enabling adjustments and optimization of implant positioning. Previous studies have largely focused on radiological outcomes—such as cup inclination and anteversion, femoral offset, and leg length discrepancy—with mixed findings. Some evidence suggests fluoroscopy may enhance component positioning and reduce dislocation risk [1], while others report no significant differences compared with conventional techniques [2,3]. However, revision for malpositioning is rare, and most studies evaluating the association between revision risk and use of fluoroscopy lack sufficient power to draw definitive conclusions [4,5].

While (radiological) precision is important to orthopedic surgeons, patient-centered outcomes such as postoperative function, complication rates, and revision rate are paramount. To the best of our knowledge, the literature is lacking a large registry study on this topic. We aimed to investigate the association between use of fluoroscopy and risk of revision in primary THA, using data from the Dutch Arthroplasty Register (LROI).

## Methods

### Data sources

Nationwide data from the Dutch Arthroplasty Register (LROI) was used. The LROI registers all arthroplasties in the Netherlands, collecting patient and procedure characteristics (e.g., sex, age, body mass index [BMI], American Society of Anesthesiologists Physical Status [ASA] class, surgical approach, implant type, and brand) at the primary procedure, as well as information regarding revision surgery (e.g., type of revision, reason for revision, survival time) and mortality [6]. THAs have been registered since the start of the registry in 2007. However, the use of modern techniques such as fluoroscopy, navigation, patient-specific instrumentation, and robotic assistance has only been registered since 2022. Data was reported using the STROBE guidelines.

### Data collection

In the Netherlands, 99% of the registered primary THAs in which fluoroscopy was used were performed using the direct anterior approach: the supine position of the patient enables use of the fluoroscope. Hence, we included only THAs performed via the direct anterior approach (DAA-THA).

We included all primary DAA-THAs reported in 2022–2024. Fluoroscopy procedures performed in a hospital with limited exposure (< 10 fluoroscopy-procedures registered during the study period) were excluded, to eliminate a potential learning curve effect. We created 2 groups: fluoroscopy (intervention) and conventional surgery (control). Conventional surgery was defined as a DAA-THA without use of fluoroscopy. Missing data per variable wase categorized separately and not included in the analyses (range: 0% to 1.3% per variable). Procedures with unknown or missing fluoroscopy registrations were excluded.

### Statistics

We created categories for continuous variables (e.g., age < 60 years, 60–75 years, ≥ 75 years) or used existing categories (e.g., ASA class) and reported absolute numbers as well as proportions (%) (see Table 1). Groups were compared using chi-square tests. Reasons for revision were reported and analyzed similarly (see Table 2). Chi-square tests were applied for most comparisons; however, in the case of 5 or less events per cell, we used Fisher’s exact test to determine statistical significance. Survival time was calculated as the time from primary THA to first revision for any reason, death of the patient, migration, or the end of the study period (January 1, 2025). We estimated crude cumulative incidence of revision, in which death was considered a competing risk, using the Aalen–Johansen estimator, which provides the absolute probability of the event over time while accounting for competing risks [7]. Rates of revision for any reason (%) at 6 months, 1, 2, and 2.5 years were reported with 95% confidence intervals (CI).

**Table 1:** Patient and procedure characteristics of primary total hip arthroplasties via the direct anterior approach performed between 2022 and 2024 in the Netherlands. Values are count (%)

Item	Fluoroscopy	Conventional	Total
	n = 18,299	n = 31,579	n = 49,878
Sex **^a^**			
Female	11,724 (64)	20,141 (64)	31,865 (64)
Male	6,568 (36)	11,436 (36)	18,004 (36)
Age **^a^**			
< 60	2,989 (16)	5,298 (17)	8,287 (17)
60–74	8,752 (48)	15,943 (51)	24,695 (50)
≥ 75	6,558 (36)	10,337 (33)	16,895 (34)
ASA class **^a^**			
I	2,632 (14)	4,592 (15)	7,224 (15)
II	10,826 (59)	19,682 (62)	30,508 (61)
III–IV	4,831 (26)	7,259 (23)	12,090 (24)
BMI			
Underweight (< 18.5)	167 (0.9)	260 (0.8)	427 (0.9)
Normal w. (18.5–25)	6,789 (37)	11,606 (37)	18,395 (37)
Overweight (25–30)	7,522 (41)	12,925 (42)	20,447 (42)
Obesity (30–40)	3,596 (20)	6,068 (20)	9,664 (20)
Morbid obesity (> 40)	112 (0.6)	190 (0.6)	302 (0.6)
Diagnosis **^a^**			
Osteoarthritis	16,349 (89)	28,562 (90)	44,911 (90)
Fracture	997 (5.4)	1,329 (4.2)	2,326 (4.7)
Dysplasia	176 (1.0)	398 (1.3)	574 (1.2)
Osteonecrosis	350 (1.9)	613 (1.9)	963 (1.9)
Non-osteoarthritis	427 (2.3)	677 (2.1)	1,104 (2.2)
Charnley score **^a^**			
A	6,962 (38)	12,066 (38)	19,028 (38)
B1	5,334 (29)	9,987 (32)	15,321 (31)
B2	4,296 (24)	7,152 (23)	11,448 (23)
C	393 (2.2)	613 (2.0)	1,006 (2.0)
N/A (non-OA)	1,135 (6.3)	1,584 (5.0)	2,719 (5.5)
Previous surgeries of affected hip			
Yes	381 (2.1)	631 (2.0)	1,012 (2.0)
No	17,918 (98)	30,948 (98)	48,866 (98)
Smoking **^a^**			
Yes	1,675 (9.3)	2,724 (8.7)	4,399 (8.9)
No	16,306 (91)	28,598 (91)	44,904 (91)
Fixation **^a^**			
Cemented	2,342 (13)	2,393 (7.6)	4,735 (10)
Cementless	13,484 (74)	25,089 (80)	38,573 (78)
Hybrid with cemented			
femur	1,472 (8.1)	3,787 (12)	5,259 (11)
acetabulum	926 (5.1)	247 (0.8)	1,173 (2.4)

a Significant difference, P < 0.05 (chi-square test)

**Table 2:** Reasons for revision after primary total hip arthroplasties via the direct anterior approach performed between 2022 and 2024 in the Netherlands. Values are count (%)

Reason for revision	Fluoroscopy	Conventional
	n = 18,299	n = 31,579
Infection	95 (0.5)	188 (0.6)
Wear inlay	2 (0.0)	2 (0.0)
Periprosthetic fracture	67 (0.4)	121 (0.4)
Dislocation	35 (0.2)	75 (0.2)
Loosening femoral component	29 (0.2)	60 (0.2)
Loosening acetabular component	17 (0.1)	30 (0.1)
Periarticular ossifications	3 (0.0)	2 (0.0)
Malalignment	25 (0.1)	55 (0.2)
Symptomatic metal-on-metal	1 (0.0)	0 (0.0)
Girdlestone	11 (0.1)	4 (0.0)
Other	26 (0.1)	44 (0.1)
Total number of revisions	245 (1.3)	505 (1.6)

A multivariable Cox proportional hazard analysis was performed, adjusted for BMI, fixation, and previous surgeries, entered as categorical variables as shown in Table 1. Hazard ratios were reported, including CI. Proportionality hazard (PH) assumption was checked by Schoenfeld residuals for all adjustment variables and the main indicator (fluoroscopy), and the assumption of proportionality was met. As the variables ASA class, age, and diagnosis showed violations of the PH assumption, they were added to the model as strata.

For all tests, a 2-sided significance level of 0.05 was used. Results were reported as revision rates (%) or hazard ratio (HRs) with CI. SPSS (28.0.1.1; IBM Corp, Armonk, NY, USA) was used for the statistical analyses.

### Ethics, data sharing plan, funding, use of AI, and disclosures

The scientific advisory committee of the LROI approved this study. As this study used anonymized registry data, ethical approval was not required [8]. The data was registered confidentially with patient consent and processed in compliance with the regulations of the LROI governing research on registry data. Sharing of data is not permitted by the LROI due to privacy regulations. No funding was received. AI tools were not used. The authors declared no conflicts of interest [7]. Complete disclosure of interest forms according to ICMJE are available on the article page, doi: 10.2340/17453674.2026.45966

## Results

**Figure 1:**
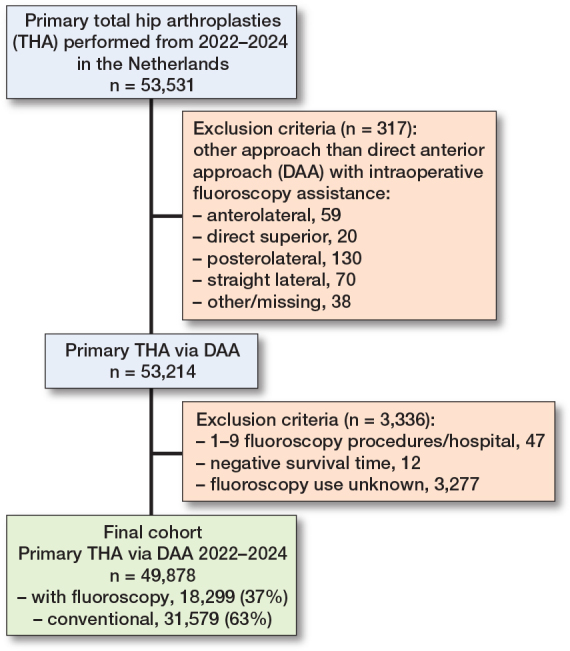
Flowchart of included procedures.

During the study period, 53,214 primary DAA-THAs were performed in 68 Dutch hospitals (Figure 1). 46/68 (67%) hospitals used fluoroscopy at least once. In 13 hospitals fluoroscopy was used in < 10 procedures, hence these procedures were excluded from the analysis (n = 47). 317 THAs with fluoroscopy using other approaches than DAA were registered and were excluded (Figure 1). 3,277 procedures (6%) showed unknown or missing fluoroscopy registrations and were also excluded from the analysis. Thus, 49,878 primary THAs were included in this study (Figure 1).

In general hospitals, 40% of DAA-THAs were performed with fluoroscopy assistance, while private hospitals used fluoroscopy less frequently (27%).

### Patient characteristics

Mean age was 69 years (median 70, range 10–101). Mean follow-up was 1.4 years (median 1.4, range 0–3).

The fluoroscopy group consisted of 18,299 procedures (37%) and the conventional group of 31,579 (63%). The patients in the fluoroscopy group were generally older, with higher ASA classes. Smoking was more frequent in the fluoroscopy group than in the conventional group. In the fluoroscopy group, cemented fixation was more frequent, as well as reversed hybrid fixation (cemented acetabulum). Fluoroscopy was more often used in patients with fracture as primary diagnosis. The conventional group showed a higher frequency of cementless fixation, as well as hybrid fixation (cementation of the femoral stem). Sex distribution was similar between the fluoroscopy and conventional group, as well as BMI, and previous surgeries of the affected hip (Table 1).

245 revisions (1.3%) were performed in the fluoroscopy group and 505 (1.6%) in the conventional group. Reasons for revision were comparable between the groups without any significant differences (Table 2).

### Survival

**Figure 2:**
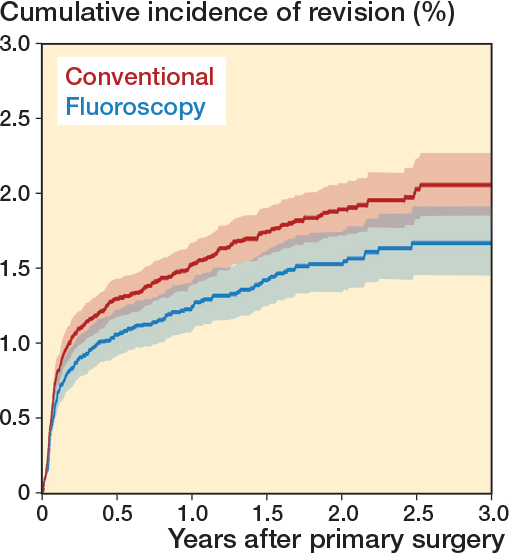
Cumulative incidence of revision after primary total hip arthroplasty in the Netherlands (2022–2024).

The competing risk analysis showed no significant differences in early unadjusted revision rates of fluoroscopy vs conventional primary THA. The cumulative incidence of revision after 2.5 years follow-up was 1.7% (CI 1.5–1.9) with fluoroscopy vs 2.0% (CI 1.8–2.3) without fluoroscopy (Table 3, Figure 2).

**Table 3:** Cumulative incidence of revision of primary total hip arthroplasties via the direct anterior approach performed between 2022 and 2024 in the Netherlands

Time	Fluoroscopy		Conventional	
	% (CI)	n at risk	% (CI)	n at risk
6 months	1.1 (0.9–1.2)	14,817	1.3 (1.2–1.4)	25,288
1 year	1.3 (1.1–1.4)	11,630	1.5 (1.4–1.7)	18,751
2 years	1.5 (1.4–1.8)	5,445	1.9 (1.7–2.1)	7,320
2.5 years	1.7 (1.5–1.9)	2,455	2.0 (1.8–2.3)	3,530

CI = 95% confidence interval.

The multivariable analysis showed a significantly decreased risk of revision for use of fluoroscopy, compared with conventional surgery, after adjustment for BMI, previous surgeries, and fixation (stratified for age, ASA class, and diagnosis): HR 0.8 (CI 0.7–0.9) (Table 4).

**Table 4:** Uni- and multivariable regression analysis of primary total hip arthroplasties via the direct anterior approach performed between 2022 and 2024 in the Netherlands

Type	Crude	Adjusted
	hazard ratio (CI)	hazard ratio (CI) ^a^
Fluoroscopy	0.8 (0.7–0.95) **^b^**	0.8 (0.7–0.9) **^b^**
Conventional	1.0	1.0

a Adjusted for previous surgeries, fixation, body mass index, stratified for age, American Society of Anesthesiologists Physical Status classification, diagnosis (osteoarthritis/non-osteoarthritis).

b Significant difference, P < 0.05

CI = 95% confidence interval.

A sensitivity analysis was performed to assess the impact of fixation on revision rate. When analyzing cemented and cementless fixation as separate subgroups, the results remained comparable with the overall multivariable analysis, showing a lower revision risk for the fluoroscopy group. Cemented fixation was less common than cementless fixation (see Table 1). The difference did not remain statistically significant when exclusively analyzing procedures with cemented fixation.  

## Discussion

We aimed to determine the association between the use of fluoroscopy and short-term revision risk following primary THA via the DAA. We showed a significantly lower risk of short-term revision after use of fluoroscopy during primary THA, when compared with conventional surgery (1.7% [CI 1.5–1.9] vs 2.0% [CI 1.8–2.3], adjusted HR 0.8, CI 0.7–0.9). However, this disappeared when examining only cemented hips. Reasons for revision were similar.

Crude cumulative incidence of revision did not show any significant differences between fluoroscopy and conventional surgery, but the patient populations were not comparable: in general, fluoroscopy was used in more complex patients with higher age and ASA class. After adjustment, the fluoroscopy group showed a significantly lower risk of revision compared with the conventional group. It is possible that surgeons specifically opted to use fluoroscopy in more complex patients. However, it is more likely that the use of fluoroscopy is bound to hospital policy, which also may influence the likelihood of being revised (e.g., private or public hospitals). Due to the privacy regulations of the LROI, we could not compare different hospitals or hospital types to further investigate this distribution and possible in-hospital variation.

The goal of fluoroscopy is to optimize implant positioning and minimize the risk of malalignment-related complications. When evaluating the short-term revision risk, one could hypothesize that there might be fewer revisions due to dislocation and periprosthetic fracture (PPF) in the fluoroscopy group, which we did not find.

No prior nationwide registry studies have been performed on the use of fluoroscopy. The majority of existing publications on fluoroscopy reported mainly on radiographic outcome measures (e.g., acetabular component positioning, leg length discrepancy, and offset difference) [1-3]. A recent systematic review and meta-analysis [5] reported no differences in radiographic outcomes, complications, and re-interventions between fluoroscopy and conventional surgery. The authors reported longer operating times for fluoroscopy, a difference of approximately 7 min (P < 0.001) compared with conventional surgery. This review was based on non-randomized studies observational studies. No randomized controlled trials have been published on this topic. It is important to note that most studies used data from experienced surgeons, who might not need the radiological feedback as much as the less experienced surgeons. Both DAA-THA and the interpretation of fluoroscopy are subject to a substantial learning curve [7,9-11]. This fact must be taken into account while interpreting the results of the current study, which entails data from surgeons of all experience levels. Stratification of experience levels was not possible, as this was not defined in the current dataset. Surgeons in their early learning curve may benefit most from fluoroscopy.

One of the theoretical disadvantages of fluoroscopy would be radiation exposure [5]. Prior studies concluded that radiation exposure of both surgeons and patients during DAA-THA is negligible [12-14]. The actual radiation time of an experienced surgeon will be very minimal, needing only a minimal amount of images to confirm adequate implant positioning and leg length.

Kirchner et al. (2022) evaluated the cost-effectiveness of fluoroscopy using a break-even model. They concluded that fluoroscopy would be cost effective with the prevention of 1 aseptic revision out of 385 primary THAs [4].

### Limitations

First, only revisions are recorded in the registry: reoperations without exchange or removal of implant components were not included in the analysis; for example: periprosthetic fracture (PPF) treated with open reduction and internal fixation (ORIF) without stem exchange, or dislocation treated with closed reduction. The number of failed or complicated implants may therefore be underestimated, and important outcome measures such as dislocation cannot be fully assessed. Second, there may be residual obscuring of the results due to unavailable variables: hospital type and code were not provided, as the combination with other variables made these hospital data potentially identifiable. Third, due to the observational design of our study, no assumptions can be made regarding causality. Fourth, in the current study, we could not differentiate between levels of surgeon experience, and therefore it is possible that the benefit of fluoroscopy in less experienced surgeons is underestimated. Lastly, the LROI started registering fluoroscopy in 2022, providing relatively low numbers and a short follow-up period. Potential effects of variation in component positioning may result in possible differences in survival due to wear or loosening of components; however, these are not detected within our currently limited follow-up. Therefore, this study was focused on short-term results. Also, the current study could not stratify for surgeon experience. It would be insightful to investigate this in the future, as fluoroscopy might be of greater benefit to less experienced surgeons in their early learning curve, compared with more experienced surgeons. Furthermore, registration of resident training could provide insights, as we believe fluoroscopy could be useful for teaching purposes.

### Conclusions

We showed that the use of fluoroscopy during primary THA using the DAA is associated with a significantly lower short-term risk of revision in the Netherlands. The data showed no major differences in revision due to malalignment and periprosthetic fractures. Revision rates were low in both groups (1.7 vs 2.0% at 2.5 years’ follow-up) and clinical differences were small.

## Notes

Authors contributed to (1) study design and study protocol, (2) gathered data, (3) data analysis, (4) initial draft, and (5) final draft. MCV, WPZ, and RMP contributed to 1–5; LvS contributed to 2, 3, and 5; PvV contributed to 3–5; PFD and JK contributed to 1, 4, and 5.

The authors would like to thank their local statistician N. Veeger for his assistance in the statistical analyses.

Handling co-editors: Keijo Mäkelä and Philippe Wagner

*Acta* thanks Sarunas Tarasevicius and other anonymous reviewers for help withpeer review of this manuscript.
